# Quantitative Analysis Method and Correction Algorithm Based on Directivity Beam Pattern for Mismatches between Sensitive Units of Acoustic Dyadic Sensors

**DOI:** 10.3390/s23125709

**Published:** 2023-06-19

**Authors:** Lingmeng Yang, Zhezheng Zhu, Wangnan Chen, Chengchen Gao, Yilong Hao, Zhenchuan Yang

**Affiliations:** School of Integrated Circuits, Peking University, Beijing 100871, China; lm_yang16@pku.edu.cn (L.Y.); triplez@pku.edu.cn (Z.Z.); chenwn@stu.pku.edu.cn (W.C.); gaocc@pku.edu.cn (C.G.); haoyl@pku.edu.cn (Y.H.)

**Keywords:** acoustic dyadic sensor, mismatch, quantitative analysis, correction algorithm

## Abstract

Acoustic dyadic sensors (ADSs) are a new type of acoustic sensor with higher directivity than microphones and acoustic vector sensors, which has great application potential in the fields of sound source localization and noise cancellation. However, the high directivity of an ADS is seriously affected by the mismatches between its sensitive units. In this article, (1) a theoretical model of mixed mismatches was established based on the finite-difference approximation model of uniaxial acoustic particle velocity gradient and its ability to reflect the actual mismatches was proven by the comparison of theoretical and experimental directivity beam patterns of an actual ADS based on MEMS thermal particle velocity sensors. (2) Additionally, a quantitative analysis method based on directivity beam pattern was proposed to easily estimate the specific magnitude of the mismatches, which was proven to be useful for the design of ADSs to estimate the magnitudes of different mismatches of an actual ADS. (3) Moreover, a correction algorithm based on the theoretical model of mixed mismatches and quantitative analysis method was successfully demonstrated to correct several groups of simulated and measured beam patterns with mixed mismatches.

## 1. Introduction

Sound signal is a very important information resource, and accurate measurement of sound signal is of great value in daily life and industrial production fields. Measurement of directionality of sound field is to use the directivity of acoustic sensors or detection systems to obtain the sound signal in a certain direction, while the sound in other directions is regarded as noise and reduced to the minimum, thus improving the signal-to-noise ratio of the acoustic detection system [1]. Acoustic sensors are the core parts of acoustic detection systems, which directly determine the quality of the picked-up sound signal. High directivity acoustic sensors play an important role in fields, such as sound source localization [2], noise cancellation [1,3], etc.

### 1.1. Acoustic Dyadic Sensors (ADSs)

According to the Taylor series expansion theory of sound pressure [4], complete sound field information includes sound pressure (scalar, zero-order spatial partial derivative), sound pressure gradient or acoustic particle velocity (vector, first-order spatial partial derivative), second-order sound pressure gradient or particle velocity gradient (dyadic, second-order spatial partial derivative) and higher order information. Additionally, the higher the order of the measured sound field information, the higher the directivity of the acoustic detection system [5,6,7,8,9,10,11], so as to meet the application requirements.

Depending on the order of the measured physical quantity, there are several types of acoustic sensors. A scalar acoustic pressure sensor (APS, e.g., microphone) is omnidirectional (directivity of order zero) and usually used as arrays to achieve directivity [12]. An acoustic vector senor (AVS, e.g., Microflown [13]) has an “8” shape directivity [14] (directivity of order one) and the array composed of AVSs has fewer sensors and smaller volume than APSs to realize the same directionality. An acoustic dyadic sensor [4] (ADS) is a new type of acoustic sensor with higher directivity (directivity of order two) than APSs (order zero) and AVSs (order one), which can measure the second-order physical quantities of the sound field at a single point.

The directivity index of an ADS with only one uniaxial acoustic particle velocity gradient channel ($\partial v_{x}/\partial x$) is 6.99 dB [15], which is nearly 1 dB (about 16.7%) higher than that of a complete triaxial acoustic vector sensor with four channels ($p,v_{x},v_{y},\mathrm{and}v_{z}$). Moreover, as long as a uniaxial particle velocity gradient channel is added to the triaxial acoustic vector sensor, the maximum directivity index of the acoustic sensor can be increased from 6.0 dB to 9.5 dB [16] (about 58.3%). Because of the high directivity of ADSs, which benefits from the ability to measure the higher quantities of the sound field at a single point, researchers consider putting ADSs into arrays to increase the directivity of arrays [17,18,19] without increasing the size of the aperture of the arrays [16].

### 1.2. Impact of Mismatches between Sensitive Units

The high directivity of an ADS depends on the accurate measurement of the second-order physical quantities of the sound field (second-order sound pressure gradient or acoustic particle velocity gradient) at its acoustic center. At present, the second-order physical quantities of sound field are usually measured using the finite-difference approximation method [4,7,20,21,22,23,24,25,26,27], so an ADS is composed of zero-order or first-order sensors. These lower-order sensors are the differential units or sensitive units of the ADS.

According to the type of sensitive units, ADSs can be divided into two types: sound pressure type and particle velocity type. Sound pressure type ADSs, composed of APSs, measure the second-order sound pressure gradient after twice finite-difference approximation of sound pressure [22]. Particle velocity type ADSs, composed of AVSs, measure the acoustic particle velocity gradient after once finite-difference approximation of acoustic particle velocity [23,24,25,26,27].

Finite-difference approximation method is an approximate measurement method, which has measurement errors. The measurement error is greatly influenced by the consistency of the differential units. Therefore, the high directivity of an ADS is extremely dependent on the consistency of its sensitive units.

When there is a mismatch between sensitive units, the measurement error of the second-order physical quantity at the acoustic center of the ADS increases [4], the directivity beam pattern at a specific frequency deviates from the ideal shape [23,24], the directivity of the ADS decreases [3], and the working frequency band of the ADS narrows [4,25]. Therefore, in order to ensure the high directivity of the ADS, it is of great significance to reduce the mismatch between sensitive units.

### 1.3. Reported Mismatch Analysis and Correction Methods

Before reducing the mismatch, it is necessary to understand the mismatch through analysis. According to the reported literatures [3,4,23,25], the mismatches of an ADS’s sensitive units can be divided into three types: amplitude mismatch, phase mismatch and axial mismatch.

Silvia [4] examined the effects of finite-difference approximations on the estimation of the first-order pressure gradient and the second-order pressure gradient (realization both by pressure sensors and accelerometers). Temporal Fourier transforms were used to derive the error formula caused by amplitude mismatch and phase mismatch. Additionally, the influence of amplitude mismatch on frequency response was quantitatively analyzed when there is no phase mismatch. The dB error in the frequency domain, resulting from estimating the complete sound field information by means of replacing the Taylor series by a Taylor polynomial of degree 2, was discussed by showing the ambiguity in the beam patterns. Additionally, a multi-channel filtering approach to directional sensors was proposed to steer the beam of the directional sensor to have maximum sensitivity in the looking direction by selecting appropriate weight combinations.

Aubauer et al. [3] reported that the sensitivity mismatch of the first-order gradient microphones, which are used to form a second-order gradient microphone, lower the directivity of the sound pressure type ADS. For a given realistic microphone sensitivity difference of 3 dB, below 500 Hz, the directivity of the ADS is close to that of the first-order gradient microphones, so that almost no gain in directivity and no noise suppression is achieved. An electronic circuit using long-time averaging method was used to enable precise sensitivity matching. As a result, the microphone sensitivity is balanced to a maximum deviation of ±0.5 dB in the dominating frequency band.

Yang et al. [23] verified the impact of amplitude mismatch and phase mismatch on the directivity of underwater acoustic velocity gradient sensors through simulations. The directivity functions and directivity beam patterns of particle velocity gradient with amplitude mismatch and phase mismatch were given separately. The simulation results showed that the beamwidth of the directivity pattern of velocity gradient will grow bigger with increase in amplitude mismatch and the whole directivity will gradually degenerate from directivity of order two to directivity of order one. When there is phase mismatch, the directivity beam pattern will become asymmetric.

Sun et al. [25] studied the axial mismatches between pairs of sensitive units of underwater acoustic velocity gradient sensors combined with one pressure sensor and six biaxial vector sensors. The formulas of measurement errors of particle velocity gradients measured by the particle velocity type ADS were derived while the axial mismatch exists. Additionally, the relationship among measurement errors, axial mismatches, azimuth angles of sound sources, and the frequency range of the underwater acoustic velocity gradient sensor were discussed. It showed that the frequency range of acoustic velocity gradient sensors will be narrowed with increasing axial mismatches for the fixed permissible error and sensitive units’ spacing.

### 1.4. Significance of This Study

The above reported literatures have laid a foundation and provided ideas for analyzing the mismatch problem of sensitive units of ADSs. Different types of mismatches were put forward and analyzed which is helpful to improve the understanding of each type of mismatch. In addition, for reducing the mismatches to ensure the high directivity of ADSs, the multi-channel filtering approach proposed by Silvia [4] can help to ignore the influence of mismatches to some extent from the point of view of signal processing. Additionally, the electronic circuit using the long-time averaging method proposed by Roland et al. [3] can reduce the influence of amplitude mismatch in a specific frequency range on the high directivity of ADSs.

However, the actual situation is complicated, and various types of mismatches may exist at the same time. Moreover, the above two methods to reduce the influence of mismatches do not make full use of the directivity beam pattern of the sensor, which can most intuitively show the sensor’s performance of pointing out the direction of sound propagation. Following are the important aspects of this study:

- A theoretical model of mixed mismatches is established to discuss and analyze the situation when all types of mismatches exist at the same time, which makes up for the lack of discussion and analysis of mixed mismatches in the reported literatures.
- A quantitative analysis method based on directivity beam pattern is proposed for analyzing the mismatches between the sensitive units of ADSs. It can help the designer of an ADS conveniently judge what type of mismatch exists in the designed sensor from the directivity beam pattern obtained from the experimental measurement, and easily estimate the specific magnitude of this type of mismatch, so as to better find the shortcomings in the original design scheme and improve it.
- A correction algorithm for the mismatches between sensitive units of ADSs is proposed, according to the theoretical model of mixed mismatches and the quantitative analysis method based on directivity beam pattern. It successfully corrects the directivity beam pattern with mixed mismatches obtained from simulation and experiment, which verifies the correctness and practicability of the theoretical model and the quantitative analysis method, and also provides a way for ADSs with mismatches to continue to play their high directivity advantages.

## 2. Theoretical Model of Mixed Mismatches

In this section, the theoretical model of mixed mismatches is established based on the finite-difference approximation model of uniaxial acoustic particle velocity gradient. Then, the change in the directivity beam pattern of an ADS with only one type of mismatch is deduced based on the theoretical model of mixed mismatches, and the influence of various mismatches on directivity beam pattern of an ADS is summarized. Thereafter, the change in directivity beam pattern in the presence of mixed mismatches will be analyzed.

### 2.1. Finite Difference Model of Uniaxial Acoustic Particle Velocity Gradient

**Figure 1:**
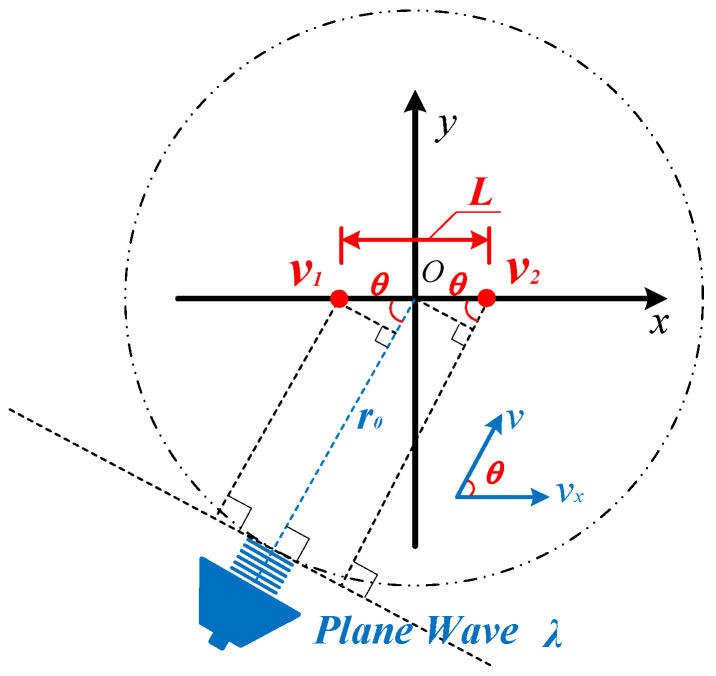
Schematic diagram of the finite-difference model of uniaxial particle velocity gradient.

As is shown in Figure 1, for the plane wave emitted by a sound source propagating in 2D space with the wave length λ, the particle velocities corresponding to two points with a spacing of L in the sound field space are, respectively, $v_{1}$ and $v_{2}$. The angle between the propagation direction of the plane wave and the positive direction of *x* axis is θ. In the process of rotation, the sound source always keeps a distance of $r_{0}$ from the origin $O\left(0,0\right)$ (that is, the center of the two points with a spacing of L in the sound field).

For harmonic plane waves, the *x* axis components of the acoustic particle velocity of the two points with a spacing of L in Figure 1, can be written as follows [28]:

(1) $$v_{1x}=\frac{p_{0}}{\rho_{0}c_{0}}e^{-j\frac{2\pi f}{c_{0}}\left(r_{0}-L\cos\theta /2\right)}\cdot \cos\theta$$

(2) $$v_{2x}=\frac{p_{0}}{\rho_{0}c_{0}}e^{-j\frac{2\pi f}{c_{0}}\left(r_{0}+L\cos\theta /2\right)}\cdot \cos\theta$$

where, $p_{0}$ is the amplitude of the incident sound wave, $\rho_{0}$ is the density of medium, $c_{0}$ is the sound speed of the medium, and f is the sound wave frequency which equals to $c_{0}/\lambda$.

According to the finite-difference approximation method, when the spacing L of the two points is much smaller than the wavelength λ (L≪λ), the acoustic particle velocity gradient at the origin point $O\left(0,0\right)$ can be estimated by the following Equation [23]:

(3) $$\frac{\partial v_{x}}{\partial x}\approx \frac{v_{2x}-v_{1x}}{L}$$

The substitution of (1) and (2) into (3) gives,

(4) $$\frac{\partial v_{x}}{\partial x}\approx \frac{p_{0}}{\rho_{0}c_{0}L}e^{-j\frac{2\pi fr_{0}}{c_{0}}}\cos\theta \left(e^{-j\frac{\pi fL\cos\theta }{c_{0}}}-e^{j\frac{\pi fL\cos\theta }{c_{0}}}\right)$$

Equation (4) is the finite difference model of uniaxial acoustic particle velocity gradient. The acoustic particle velocity at the origin point $O\left(0,0\right)$ can be estimated as [23],

(5) $$v_{x}\approx \frac{p_{0}}{2\rho_{0}c_{0}}e^{-j\frac{2\pi fr_{0}}{c_{0}}}\cos\theta \left(e^{-j\frac{\pi fL\cos\theta }{c_{0}}}+e^{j\frac{\pi fL\cos\theta }{c_{0}}}\right)$$

Simplifying Equations (4) and (5), and considering L≪λ, we can get

(6) $$\frac{\partial v_{x}}{\partial x}\approx \frac{p_{0}}{\rho_{0}c_{0}}e^{-j\frac{2\pi fr_{0}}{c_{0}}}\cdot \frac{\pi f}{c_{0}}\cos^{2}\theta$$

(7) $$v_{x}\approx \frac{p_{0}}{\rho_{0}c_{0}}e^{-j\frac{2\pi fr_{0}}{c_{0}}}\cdot \cos\theta$$

**Figure 2:**
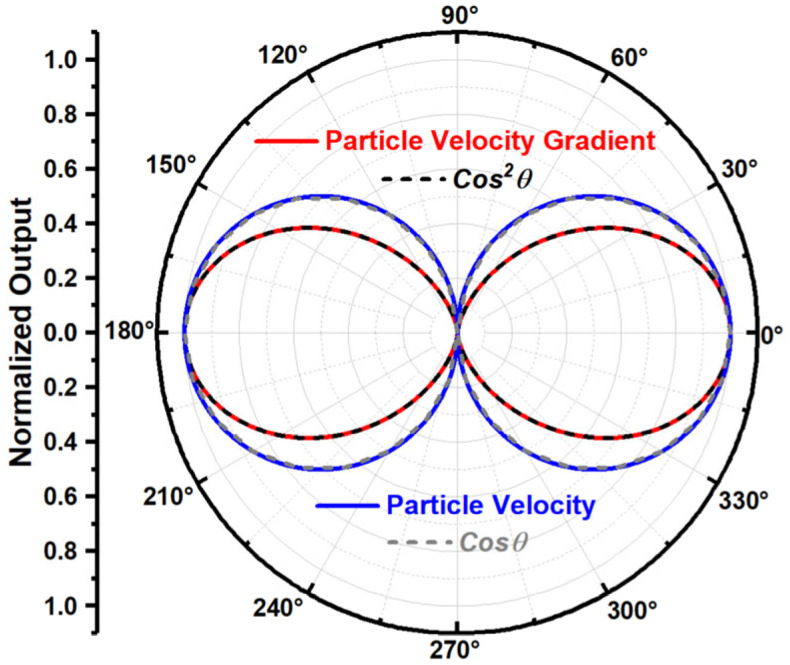
The ideal directivity beam pattern of the ADS.

Equations (6) and (7) show that, at certain frequency (L≪λ), the acoustic particle velocity gradient at the center of the two points is directly proportional to the cosine squared function of the sound wave incident angle, and the particle velocity at that point is direct proportional to the cosine function of the sound wave incident angle. The ideal directivity beam pattern of the ADS measuring this particle velocity gradient and particle velocity is shown in Figure 2.

### 2.2. Model of Mixed Mismatches between Velocity Sensitive Units

**Figure 3:**
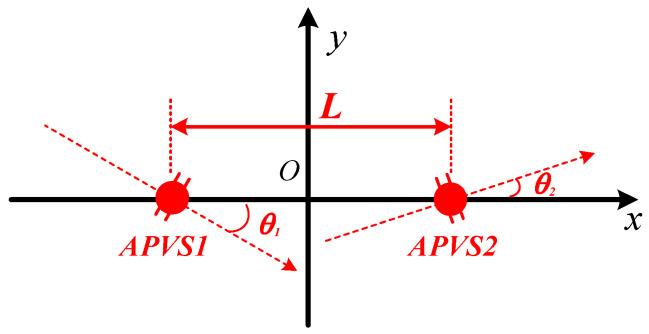
The deviation of the actual sensitive axes of the two APVSs (sensitive units of an ADS).

Consider an ADS using two actual acoustic particle velocity sensors (APVS) as its sensitive units to measure the particle velocity of the two points in Figure 1; the midpoint of the connection line between the two APVSs is also the acoustic center of the ADS. The measured *x* axis components of particle velocity at the two points may deviate from Equations (1) and (2) due to the inconsistency of the two APVSs, even under the condition when L≪λ is satisfied. The measured *x* axis components of particle velocity at the two points in Figure 1 by two actual APVSs can be written as:

(8) $$v_{1x}=A_{1}\cdot e^{-j\frac{2\pi f}{c_{0}}r_{0}}\cdot e^{j\phi_{1}}\cdot e^{j\frac{\pi fL\cos\theta }{c_{0}}}\cdot \cos\left(\theta -\theta_{1}\right)$$

(9) $$v_{2x}=A_{2}\cdot e^{-j\frac{2\pi f}{c_{0}}r_{0}}\cdot e^{j\phi_{2}}\cdot e^{-j\frac{\pi fL\cos\theta }{c_{0}}}\cdot \cos\left(\theta -\theta_{2}\right)$$

where, $A_{1}$ and $A_{2}$ are the amplitude parameters, $\phi_{1}$ and $\phi_{2}$ are the phase parameters, and $\theta_{1}$ and $\theta_{2}$ are the axial parameters. Among these parameters, $\theta_{1}$ and $\theta_{2}$ are the angles between the actual sensitive axes and the ideal sensitive axes of the two APVSs, respectively, which are shown in Figure 3.

For the ideal case, where $A_{1}=A_{2}=p_{0}/\rho_{0}c_{0}$, $\phi_{1}=\phi_{2}=0$ and $\theta_{1}=\theta_{2}=0$, Equations (8) and (9) are equal to Equations (1) and (2). In this case, it can be considered that there is no mismatch between the two sensitive units (APVS1 and APVS2 in Figure 3) of the ADS. However, when the mismatches exist, the acoustic particle velocity gradient at the acoustic center of the ADS measured using the finite-difference approximation method equals,

(10) $$\frac{\partial v_{x}}{\partial x}=\frac{e^{-j\frac{2\pi f}{c_{0}}r_{0}}}{L}\left[A_{2}\cos\left(\theta -\theta_{2}\right)e^{j\phi_{2}}e^{-j\frac{\pi fL\cos\theta }{c_{0}}}-A_{1}\cos\left(\theta -\theta_{1}\right)e^{j\phi_{1}}e^{j\frac{\pi fL\cos\theta }{c_{0}}}\right]$$

Equation (10) is the theoretical model of mixed mismatches for the particle velocity gradient measured by a particle velocity type ADS with the particle velocity sensitive units are mismatched with each other. 

### 2.3. Theoretical Directivity Beam Patterns with Single Mismatch

Equation (10) describes the estimated value of the acoustic particle velocity gradient at the acoustic center of the ADS when the amplitude, phase and axis of the two sensitive units are all mismatched. From Equation (10), the directivity beam pattern of the sensor can be obtained when there is only a single mismatch, so as to better show the influence of various types of mismatches on the directivity of the ADS.

#### 2.3.1. Beam Patterns with Amplitude Mismatch

When only amplitude mismatch exists, $A_{1}\neq A_{2}$, $\phi_{1}=\phi_{2}=0$ and $\theta_{1}=\theta_{2}=0$. Let $\mu =A_{2}/A_{1}$, then the normalized directivity function of the particle velocity gradient derived from Equation (10) can be written as:

(11) $${NDF}_{VG}\left(\theta ,\mu \right)=\cos\theta \left[\mu \cdot e^{-j\frac{\pi fL\cos\theta }{c_{0}}}-e^{j\frac{\pi fL\cos\theta }{c_{0}}}\right]$$

**Figure 4:**
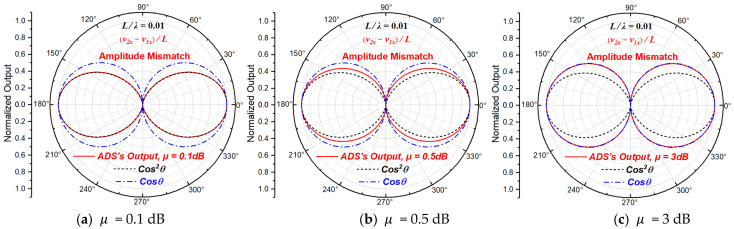
The beam patterns of the ADS with different amplitude mismatch magnitudes.

When L/λ=0.01 (L≪λ), the directivity beam patterns of the particle velocity gradient at the acoustic center of the ADS with different amplitude mismatch magnitudes are shown in Figure 4. Equation (11) is consistent with the one in the literature [23]. It is worth noting that the usage habit in the acoustic field is to use dB to represent the magnitude of amplitude mismatch, so the μ in Figure 4 is actually equal to $20\;\log_{10}\left(A_{2}/A_{1}\right)$. 

From Figure 4, it can be seen that the beam width of the directivity beam pattern of velocity gradient increases with the increase in amplitude mismatch, and the whole directivity gradually degenerates from directivity of order two to directivity of order one.

#### 2.3.2. Beam Patterns with Phase Mismatch

When only phase mismatch exists, $A_{1}=A_{2}=p_{0}/\rho_{0}c_{0}$, $\phi_{1}\neq \phi_{2}$ and $\theta_{1}=\theta_{2}=0$. Since the mismatch is relative, it will not lose the generality to let $\phi_{1}=0$ and $\phi_{2}=\phi$, then the normalized directivity function of the particle velocity gradient derived from Equation (10) can be written as:

(12) $${NDF}_{VG}\left(\theta ,\phi \right)=\cos\theta \left[e^{j\phi }\cdot e^{-j\frac{\pi fL\cos\theta }{c_{0}}}-e^{j\frac{\pi fL\cos\theta }{c_{0}}}\right]$$

**Figure 5:**
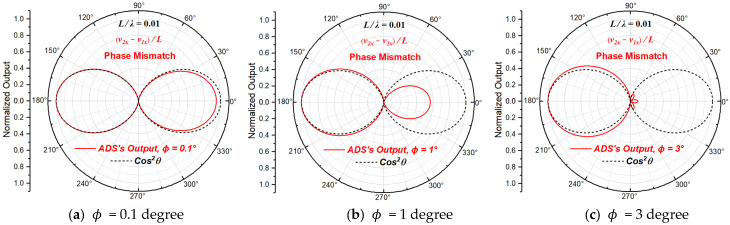
The beam patterns of the ADS with different phase mismatch magnitudes.

When L/λ=0.01 (L≪λ), the directivity beam patterns of the particle velocity gradient at the acoustic center of the ADS with different phase mismatch magnitudes are shown in Figure 5. Equation (12) is consistent with the one in the literature [23].

As is shown in Figure 5, for L/λ=0.01, the directivity of the ADS is quite sensitive to the phase mismatch of the two particle velocity sensing units, and the change in beam pattern caused by phase mismatch is more complicated compared with the one caused by amplitude mismatch. When there is phase mismatch, the directivity beam pattern becomes asymmetric, and the beam width of the larger half increases with the increase in phase mismatch, and finally the whole directivity degenerates from asymmetric directivity of order two to asymmetric directivity of order one.

#### 2.3.3. Beam Patterns with Axial Mismatch

When only axial mismatch exists, $A_{1}=A_{2}=p_{0}/\rho_{0}c_{0}$, $\phi_{1}=\phi_{2}=0$ and $\theta_{1}\neq \theta_{2}$. Since the mismatch is relative, it will not lose the generality to let $\theta_{1}=0$ and $\theta_{2}=\theta_{m}$, then the normalized directivity function of the particle velocity gradient derived from Equation (10) can be written as:

(13) $${NDF}_{VG}\left(\theta ,\theta_{m}\right)=\cos\left(\theta -\theta_{m}\right)e^{-j\frac{\pi fL\cos\theta }{c_{0}}}-\cos\theta e^{j\frac{\pi fL\cos\theta }{c_{0}}}$$

**Figure 6:**
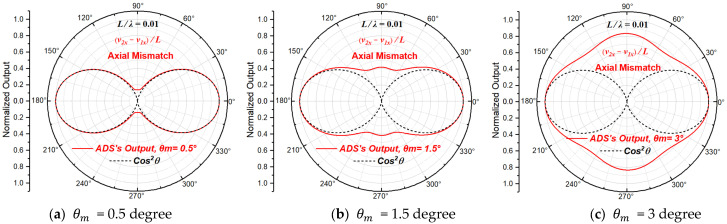
The beam patterns of the ADS with different axial mismatch magnitudes.

When L/λ=0.01 (L≪λ), the directivity beam patterns of the particle velocity gradient at the acoustic center of the ADS with different axial mismatch magnitudes are shown in Figure 6. Equation (13) is consistent with the one in the literature [25].

As illustrated in Figure 6, for L/λ=0.01, the directivity of the ADS is also sensitive to the axial mismatch of the two particle velocity sensing units, and the change in beam pattern caused by axial mismatch is complicated too. When axial mismatch exits, the concave points of the beam pattern bulges outward, and the sensitive axis direction of the beam pattern is deflected with the increase in the axial mismatch. Furthermore, the whole directivity also gradually degenerates to directivity of order one.

Summarizing the effects of the above mismatches on the directivity beam pattern of an ADS, we can get Table 1.

### 2.4. Theoretical Directivity Beam Patterns with Mixed Mismatches

**Figure 7:**
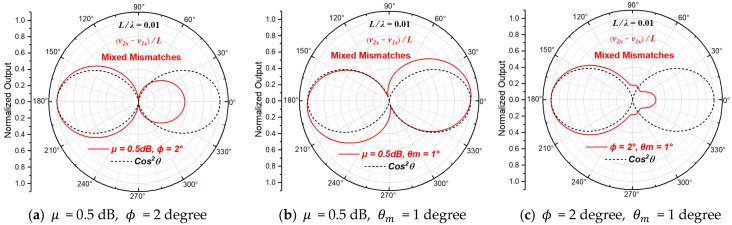
The beam patterns of the ADS with two different types of mismatches.

When there are two types of mismatches between two sensitive units at the same time, their normalized directivity beam patterns of the particle velocity gradient derived from Equation (10) are shown in Figure 7 (L/λ=0.01).

From Figure 7, we can see that (1) the increase in the beam width and the asymmetry appears at the same time when both amplitude and phase mismatches exist, (2) the increase in the beam width and the deflection of the sensitive axis appears at the same time when both amplitude and axial mismatches exist, and (3) the asymmetry and the bulge of concave points appears at the same time when both phase and axial mismatches exist.

Comparing Equations (11) and (13), we can find that both amplitude mismatch and axial mismatch will have an impact on the amplitude of the measured particle velocity gradient, so there will be mutual coupling between amplitude mismatch and axial mismatch when they exist at the same time. This can be also seen from Figure 7, in which the magnitude of the increase in beam width is larger when both amplitude and axial mismatches exist than that when only one of them exists. 

When all three types of mismatches between the two particle velocity sensitive units exist, the normalized directivity function of the particle velocity gradient derived from Equation (10) can be written as:

(14) $${NDF}_{VG}\left(\theta ,\mu ,\phi ,\theta_{m}\right)=\mu e^{j\phi }\cos\left(\theta -\theta_{m}\right)\cdot e^{-j\frac{\pi fL\cos\theta }{c_{0}}}-\cos\theta e^{j\frac{\pi fL\cos\theta }{c_{0}}}$$

**Figure 8:**
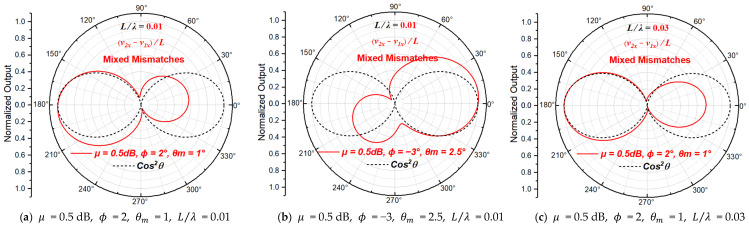
The beam patterns of the ADS with three types of mismatches.

The directivity beam patterns of the particle velocity gradient at the acoustic center of the ADS with combinations of different mismatch magnitudes are shown in Figure 8.

It can be seen from Figure 8 that the increase in the beam width, the asymmetry and the bulge of concave points or deflection of the sensitive axis appears at the same time, when all three types of mismatches between sensitive units of the ADS exist. Additionally, the change in the pattern is different for different L/λ.

### 2.5. Measured Directivity Beam Patterns with Mixed Mismatches

**Figure 9:**
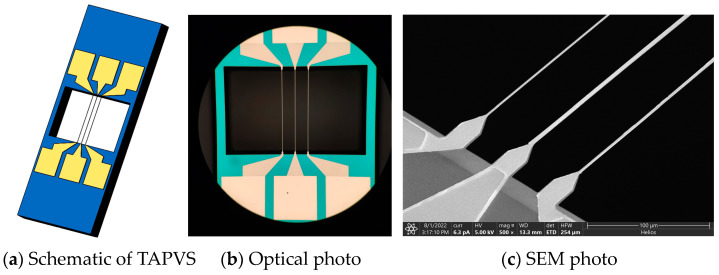
The schematic of the TAPVS chip’s structure and the photos of its thermistor cantilevers.

In our previous work [27], we proposed a compact acoustic particle velocity gradient sensor based on MEMS thermal acoustic particle velocity sensor (TAPVS) [29] chips. The MEMS TAPVS is a kind of acoustic vector senor that can directly measure the particle velocity of the sound signals [13]. The schematic of the MEMS TAPVS chip’s structure used in this article is shown in Figure 9a and the optical and SEM photos of its thermistor cantilevers are shown in Figure 9b,c. The core structures of a MEMS TAPVS chip are three thermistor cantilevers whose spacing used in this article is 50 μm. The length, width and thickness of the cantilevers are 1 mm, 2 μm and 530 nm, respectively. When there is sound incident on the three heated cantilevers, the temperature difference between the left and right cantilevers is directly proportional to the particle velocity of the sound signals [30].

**Figure 10:**
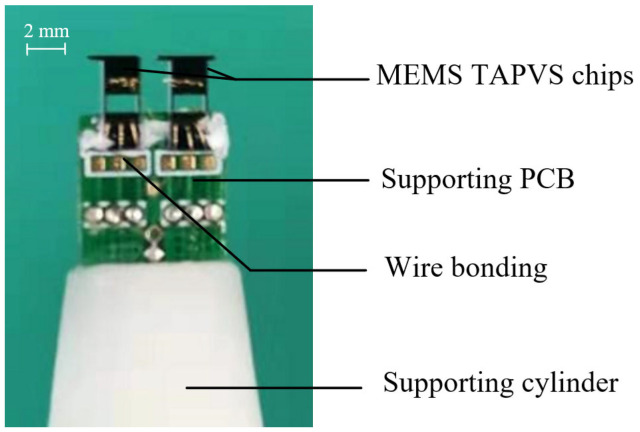
A particle velocity type ADS based on MEMS TAPVS chips.

Two MEMS TAPVS chips can be used as particle velocity sensing units to form a particle velocity type ADS, as shown in Figure 10. The spacing L of the two particle velocity sensing units of this ADS is 3.6 mm.

**Figure 11:**
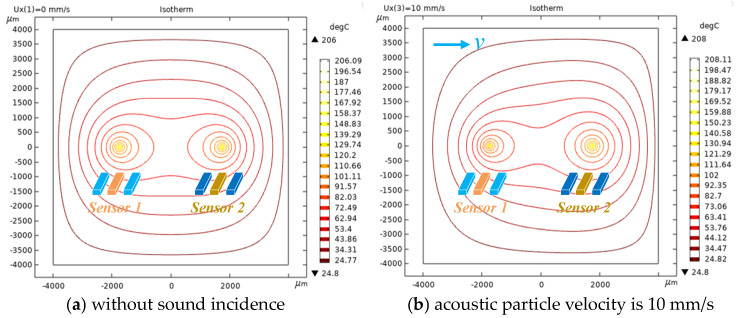
The isotherm distribution near the two TAPVS’s cantilevers.

The ADS’s 2D temperature distribution in Figure 10 is simulated by using the module of heat transfer in solids and fluids of COMSOL. The simulated geometry is set as two pairs of three-wire structures and each three-wire structure represents a TAPVS. The spacing of the wires for each TAPVS is 50 μm and the spacing of the two TAPVS is 3.6 mm. The length, width and thickness of the wires are 1 mm, 2 μm and 530 nm, respectively. The mesh is symmetrical and its size is much smaller than that of the geometric structure. The area of simulated air domain is large enough and the boundary temperature of the air domain is set to 20 °C. The isotherm distribution near the two TAPVS’s cantilevers is shown in Figure 11.

As shown in Figure 11, the temperature distributions at the left TAPVS and the right TAPVS are different. The temperature differences measured by the two TAPVS chips are directly proportional to $v_{1}$ and $v_{2}$ in Figure 1, respectively.

**Figure 12:**
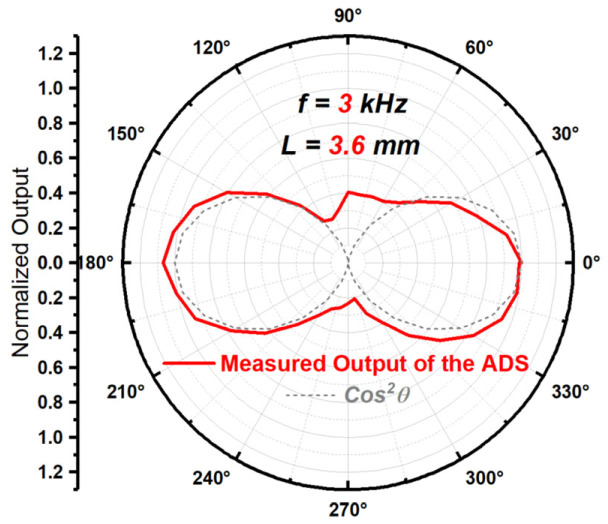
The measured directivity beam pattern of the ADS from Figure 10.

**Figure 13:**
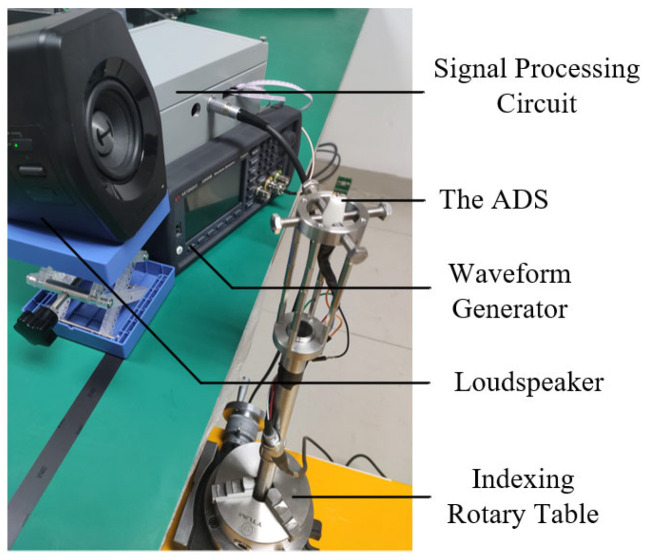
The measurement setup used to measure the beam pattern of the ADS in Figure 10.

The particle velocity gradient can be obtained by the difference between the outputs of two TAPVSs and its variation with the angle of sound incident direction θ is shown in Figure 12. The measurement setup used to obtain the measured beam pattern in Figure 12 is the same with the one in [27], which is also shown in Figure 13. Pules sound signals generated via a waveform generator is played on a loudspeaker. The incident sound wave angles received by the ADS are controlled by the indexing rotary table. The velocity signals from two TAPVSs are collected after going through the signal processing circuit with several filters and amplifiers. The output of the ADS is obtained after the two TAPVSs’ outputs go through a differential amplifier. The distance of $r_{0}$ in Figure 1 equals 0.26 m.

From Figure 12, we can find that the concave points of the beam pattern bulges outward, the beam width is slightly increased and the beam pattern has slight asymmetry. From the previous theoretical analysis using the theoretical model of mixed mismatches, such as Figure 6 and Table 1, we can see that all three types of mismatches, axial mismatch, phase mismatch and amplitude mismatch, exist between the two particle velocity sensitive units of the ADS in Figure 10.

## 3. Quantitative Analysis Method Based on Directivity Beam Pattern

As mentioned in Section 2.5, in the design process of an ADS, we can preliminarily and qualitatively judge the type of mismatch that exists in the designed ADS by comparing the measured beam pattern with the beam pattern given by the theoretical model of mixed mismatches. In this section, a quantitative analysis method based on directivity beam pattern will be proposed to easily estimate the specific magnitude of the mismatches.

### 3.1. Quantitative Analysis for Amplitude Mismatch

It can be understood from Table 1 that the main feature of the directivity beam pattern is the increase in beam width when there is only amplitude mismatch. As seen in Figure 4, the magnitude of the beam width increases when the amplitude mismatch increases. It can also be found that the ratio of the normalized directivity function when the incident angle θ is 0 degrees to that when it is 30 degrees can reflect the beam width of the ADS’s directivity beam pattern.

Therefore, we define the difference between the ratio of the ideal cosine squared beam pattern and the ratio of the beam pattern with amplitude mismatch μ as the Beam Width Increase (BWI) of an ADS. Using Equation (11), the BWI can be written as follows:

(15) $${BWI}_{VG}\left(\mu \right)=20\;\log_{10}\left(\frac{\cos^{2}0{}^{\circ}}{\cos^{2}30{}^{\circ}}\right)-20\;\log_{10}\left(\frac{{NDF}_{VG}\left(0{}^{\circ},\mu \right)}{{NDF}_{VG}\left(30{}^{\circ},\mu \right)}\right)\;=2.5-20\;\log_{10}\left(\frac{2}{\sqrt{3}}\frac{\mu \cdot e^{-j\frac{\pi fL}{c_{0}}}-e^{j\frac{\pi fL}{c_{0}}}}{\mu \cdot e^{-j\frac{\sqrt{3}\pi fL}{{2c}_{0}}}-e^{j\frac{\sqrt{3}\pi fL}{{2c}_{0}}}}\right)$$

**Figure 14:**
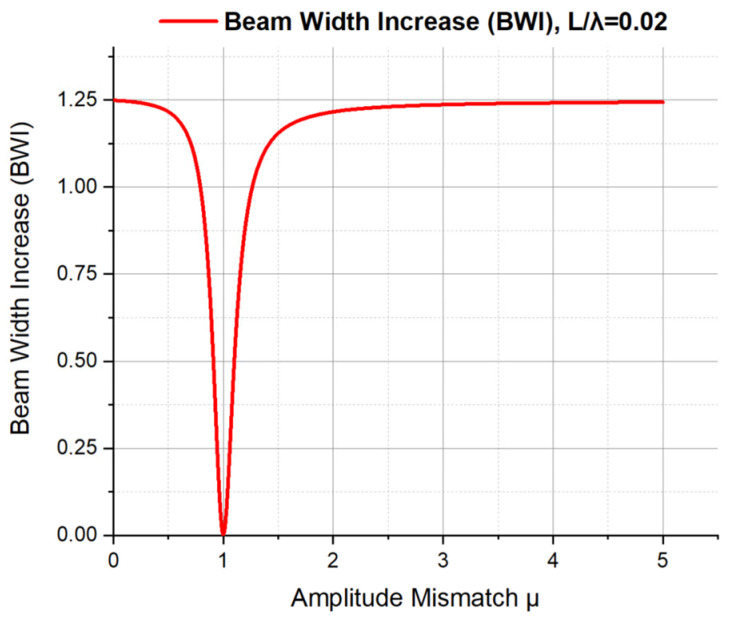
The Beam Width Increase (BWI) with the change in amplitude mismatch μ.

As seen in Equation (15), for a certain L/λ, the only variable in the expression of the BWI of an ADS is the magnitude μ of amplitude mismatch. The curve of the BWI with the change in μ is shown in Figure 14 (L/λ=0.02).

From Figure 14, we can infer the following:

1. When μ=0, the BWI equals to 1.25. It means that the second term on the right side of Equation (15) is equal to 1.25, which is the ratio of the ideal cosine beam pattern. Look back to Equation (11), when μ=0, the two exponential terms in the normalized directivity function become one, and the magnitude of the function becomes directly proportional to the cosine function of the incident angle θ (L≪λ). In other words, in this case, the directivity of order two with a cosine squared shape of an ADS has degenerated into the directivity of order one with a cosine shape.
2. When 0<μ<1, the BWI monotonically decreases from 1.25 to 0 with the increase in μ. It means that the negative influence of amplitude mismatch on directivity is decreasing. Additionally, in this case, look back to Equations (8), (9) and (11), the output amplitude of APVS2 (at the right side point) is smaller than that of APVS1 (at the left side point).
3. When μ=1, the BWI is 0. There is no amplitude mismatch in this case. Look back to Equations (8), (9) and (11), the amplitude parameters $A_{1}$ and $A_{2}$ of the two APVSs are equal to each other.
4. When μ>1, the BWI monotonically increases from 0 to 1.25. In this case, the output amplitude of APVS2 is larger than that of APVS1, and the beam width of the directivity beam pattern of velocity gradient increases with the increase in amplitude mismatch, and the whole directivity gradually degenerates from directivity of order two to directivity of order one, which has been shown in Figure 4.

### 3.2. Quantitative Analysis for Phase Mismatch

It can be also seen from Table 1 that, when there is only phase mismatch, the main feature of the directivity beam pattern is asymmetry. Additionally, the magnitude of the asymmetry increases with the increase in phase mismatch when the beam width is not so large that the shape of the beam pattern is close to the cosine squared shape, which can be seen from Figure 5.

We use the Difference of Axial Sensitivity (DAS) to quantitatively describe the asymmetry caused by the phase mismatch ϕ. Using Equation (12), the DAS of an ADS can be written as follows:

(16) $${DAS}_{VG}\left(\phi \right)=20\;\log_{10}\left(\frac{{NDF}_{VG}\left(180{}^{\circ},\phi \right)}{{NDF}_{VG}\left(0{}^{\circ},\phi \right)}\right)=20\;\log_{10}\left(\frac{1-e^{j\phi }e^{j\frac{2\pi fL}{c_{0}}}}{e^{j\phi }-e^{j\frac{2\pi fL}{c_{0}}}}\right)$$

**Figure 15:**
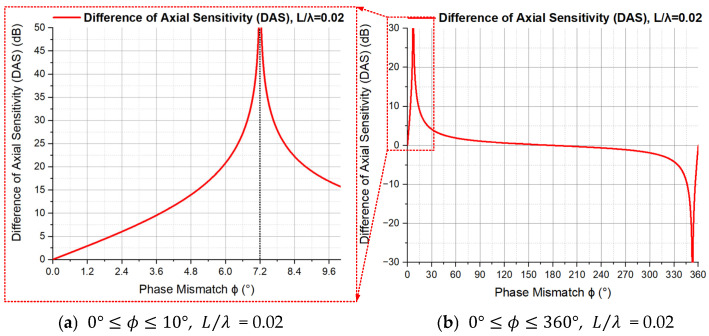
The Difference of Axial Sensitivity (DAS) with the change in phase mismatch ϕ.

As seen in Equation (16), for a certain L/λ, the only variable in the expression of the DAS of an ADS is the magnitude ϕ of phase mismatch. The curve of the DAS with the change in ϕ is shown in Figure 15 (L/λ=0.02).

From Figure 15, we can observe the following:

1. When ϕ=0°, the DAS equals to 0. There is no phase mismatch in this case.
2. When $0{}^{\circ}<\phi <\phi_{0}$, the DAS monotonically and rapidly increases from 0 to close to infinity with the increase in ϕ. It means that the asymmetry of the directivity beam pattern monotonically and rapidly increases with the increase in phase mismatch ϕ, so the high directivity of the ADS is quite sensitive to phase mismatch in this case. Here, for L/λ = 0.02, $\phi_{0}=7.2{}^{\circ}$, which is determined by ${NDF}_{VG}\left(0{}^{\circ},\phi \right)$.
3. When $\phi \approx \phi_{0}$, the DAS is close to infinity. In this case, look back to Equation (12) and Figure 5, the normalized directivity function is close to 0 when the incident angle θ is 0 degrees and the right part of the beam pattern becomes the smallest.
4. When $\phi_{0}<\phi \leq 180{}^{\circ}$, the DAS monotonically and slowly decreases to 0. In this stage, although the asymmetry of the beam pattern decreases, the directivity beam pattern of the ADS gradually degenerates from the directivity of order two with a cosine squared shape to the directivity of order one with a cosine shape, which can be seen in Figure 5.
5. When 180°<ϕ≤360°, the change in the DAS with the phase mismatch ϕ is symmetrical with the change when 0°≤ϕ≤180°. In this case, the phase relationship between APVS1 and APVS2 is opposite to the case when 0°≤ϕ≤180°.

### 3.3. Quantitative Analysis for Axial Mismatch

Table 1 also shows that, when there is only axial mismatch, the main features of the directivity beam pattern are the concave points bulge and sensitive axis direction deflected. Additionally, the magnitude of the bulge increases with the increase in axial mismatch before the deflection happens, which can be seen in Figure 6.

Therefore, we define the ratio of the normalized directivity function when the incident angle θ is 0 degrees and 90 degrees, as the Ideal Axial Ratio (IAR) of an ADS, when there is axial mismatch $\theta_{m}$. Using Equation (13), the IAR can be written as follows:

(17) $${IAR}_{VG}\left(\theta_{m}\right)=20\;\log_{10}\left(\frac{{NDF}_{VG}\left(0{}^{\circ},\theta_{m}\right)}{{NDF}_{VG}\left(90{}^{\circ},\theta_{m}\right)}\right)=20\;\log_{10}\left(\frac{\cos\theta_{m}e^{-j\frac{\pi fL}{c_{0}}}-e^{j\frac{\pi fL}{c_{0}}}}{\sin\theta_{m}}\right)$$

**Figure 16:**
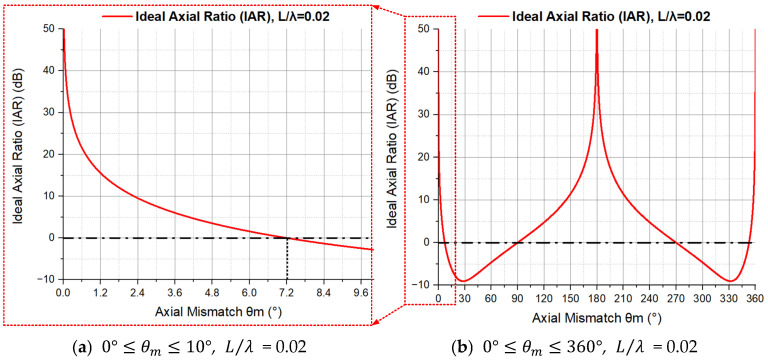
The Ideal Axial Ratio (IAR) with the change in axial mismatch $\theta_{m}$.

As seen in Equation (17), for a certain L/λ, the only variable in the expression of the IAR of an ADS is the magnitude $\theta_{m}$ of axial mismatch. The curve of the IAR with the change in $\theta_{m}$ is shown in Figure 16 (L/λ=0.02).

From Figure 16, we observe the following:

1. When $\theta_{m}=0{}^{\circ}$, the IAR equals to infinity. There is no axial mismatch in this case.
2. When $0{}^{\circ}<\theta_{m}<\theta_{m0}$, the IAR monotonically and rapidly decreases to 0 with the increase in $\theta_{m}$. Look back to Figure 6, we can find that the concave points bulge is getting bigger and the sensitive axis direction is still along the original direction during this stage. When L/λ = 0.02, $\theta_{m0}=7.2{}^{\circ}$, which is determined by ${NDF}_{VG}\left(0{}^{\circ},\theta_{m}\right)$.
3. When $\theta_{m}\approx \theta_{m0}$, the IAR equals to 0. As seen in Figure 6, the magnitude of the lateral output (when the incident angle θ of the sound wave is 90 degrees) of the ADS is almost the same with the axial output (the incident angle θ is 0 degrees), so the strong ability of the ADS to distinguish sound waves incident along the axial direction is greatly reduced.
4. When $\theta_{m0}<\theta_{m}<180{}^{\circ}$, the IAR first becomes negative, and then gradually increases with the increase in axial mismatch $\theta_{m}$. As seen in Figure 6, the sensitive axis direction of the beam pattern has been deflected and the whole directivity also gradually degenerates to directivity of order one.
5. When $180{}^{\circ}<\theta_{m}\leq 360{}^{\circ}$, the change in the IAR with the phase mismatch $\theta_{m}$ is symmetrical with the change when $0{}^{\circ}\leq \theta_{m}\leq 180{}^{\circ}$.

From the above analysis, we can see that, for a certain L/λ, the three quantitative parameters, Beam Width Increase (BWI), Difference of Axial Sensitivity (DAS) and Ideal Axial Ratio (IAR) can quantitatively reflect how the beam pattern will change when the magnitude of the mismatch reaches a specific value. These three quantitative parameters of an ADS’s directivity beam pattern are summarized in Table 2.

### 3.4. Quantitative Analysis for Measured Beam Patterns

By using the three quantitative parameters in Table 2, the designer of an ADS can conveniently judge what type of mismatch exists in the designed sensor from the directivity beam pattern obtained from the experimental measurement, and easily estimate the specific magnitude of this type of mismatch, so as to better find the shortcomings in the original design scheme and improve it.

Here, we are going to use two actual ADSs as examples to illustrate the usage of this quantitative analysis method for mismatches between the sensitive units of an ADS based on directivity beam pattern. 

One ADS is shown in Figure 10, which is a particle velocity type ADS based on MEMS TAPVS chips, similar to the one in our previous work [27]. The spacing L of the two TAPVS chips is 3.6 mm. Its directivity beam pattern was obtained at the frequency of 3 kHz, as shown in Figure 12.

As seen in Figure 12, the beam pattern is not perfect cosine squared shape. Additionally, the sensitive units’ axial mismatch of the ADS in Figure 10 is larger than that of the ADS in our previous work [27]. After carefully comparing the two ADSs, it is further found that, in the ADS in Figure 10, it is more difficult to control the sensitive axis directions of the APVS units in the process of assembling two APVS chips, due to the lack of more support from printed circuit board (PCB) compared with the ADS in our previous work [27]. Therefore, the ADS in Figure 10 has a greater probability of axial mismatch between two sensitive units.

The deviation of the results shown in Figure 12 is more serious than that in [27]. One benefit of this is that, it can be easier to illustrate the influence of mixed mismatches on the directivity of ADSs. Therefore, the result of Figure 12 is taken as an example, and the mismatch of the ADS in Figure 10 will be quantitatively analyzed using the three quantitative parameters described above, based on the beam pattern shown in Figure 12.

First, for amplitude mismatch, the actual Beam Width Increase (BWI) can be calculated by the following steps, which is also illustrated using Equation (18):

1. Calculate the ratio of the measured output data when the incident angle θ of the sound wave is 180 degrees to the measured data when θ is 150 degrees. The reason for choosing the angles (180 and 150) of the left side beam instead of the angles (0 and 30) of the right side is that the deviation of the left side beam from the ideal cosine squared shape is closer to the prediction of the theoretical model in Section 2.
2. Calculate the difference between this ratio and the ratio of the ideal cosine squared beam pattern, which is similar to Equation (15).

(18) $${BWI}_{VG\_actual}=2.5-20\;\log_{10}\left(\frac{Data\_measured\left(180{}^{\circ}\right)}{Data\_measured\left(150{}^{\circ}\right)}\right)$$

For the measured beam pattern shown in Figure 12, the ${BWI}_{VG\;actual}$ is equal to 0.0779. Substituting 0.0779 into Equation (15), the magnitude of amplitude mismatch μ can be estimated as 1.048 (0.41 dB).

Next, for phase mismatch, the actual Difference of Axial Sensitivity (DAS) can be calculated as follows:

(19) $${DAS}_{VG\_actual}=20\;\log_{10}\left(\frac{Data\_measured\left(180{}^{\circ}\right)}{Data\_measured\left(0{}^{\circ}\right)}\right)$$

For the measured beam pattern shown in Figure 12, the ${DAS}_{VG\;actual}$ equals to 0.566. Substituting 0.566 into Equation (16), the magnitude of phase mismatch ϕ can be estimated as 0.374 degrees.

At last, for axial phase mismatch, the actual Ideal Axial Ratio (IAR) can be calculated as follows:

(20) $${IAR}_{VG\_actual}=20\;\log_{10}\left(\frac{Data\_measuredt\left(0{}^{\circ}\right)}{Data\_measured\left(90{}^{\circ}\right)}\right)$$

For the measured beam pattern shown in Figure 12, the ${IAR}_{VG\;actual}$ equals to 13.257. Substituting 13.257 into Equation (17), the magnitude of axial mismatch $\theta_{m}$ can be estimated as 2.481 degrees.

The magnitude of each type of mismatch of the ADS from [27] can be estimated quantitatively in the same way, and the results are shown in Table 3.

**Figure 17:**
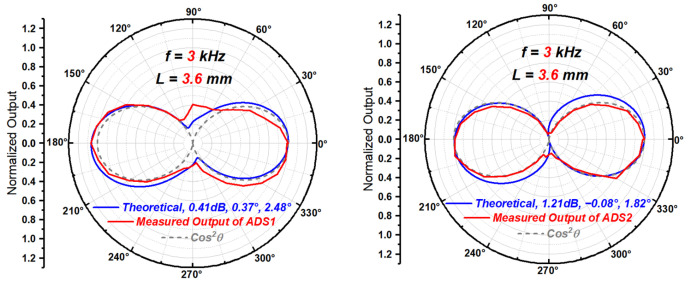
The theoretical beam patterns of ADS1 from Figure 10 and ADS2 from [27] with mismatches in Table 3.

**Figure 18:**
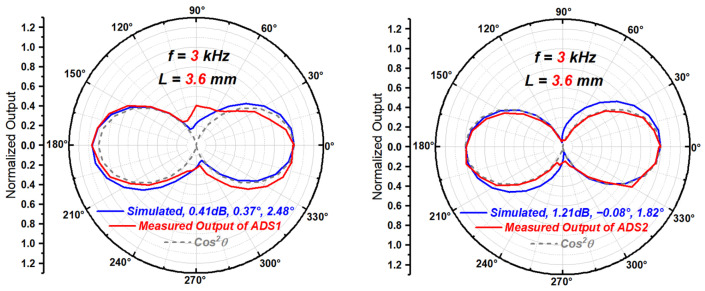
The simulated beam patterns of ADS1 from Figure 10 and ADS2 from [27] with mismatches in Table 3.

Substituting the estimated μ, ϕ and $\theta_{m}$ into Equation (14), the normalized directivity function with all three types of mismatches mixed together, the theoretical directivity beam pattern of an ADS with such mismatches can be drawn, as shown in Figure 17. The simulated results in Figure 18 can be obtained by using the pressure acoustics module of COMSOL in the frequency domain. Taking the plane wave field propagating in a 3D cylindrical pipe with a hard boundary wall as the sound field to be measured, the particle velocities at two points with a distance of 3.6 mm in the sound field are selected as the outputs of the two particle velocity sensitive units. Different mismatches can be achieved by directly adjusting the particle velocities extracted at the two points. The mesh is symmetrical and its size is much smaller than the wavelength of sound wave. The different incident directions of sound wave can be realized by adjusting the direction of the cylindrical pipe.

As seen in Figure 17 and Figure 18, the quantitative analysis method based on directivity beam pattern using the three quantitative parameters in Table 2, can approximatively reflect the mixed mismatches between the sensitive units of an ADS to some extent.

Therefore, the designer of an ADS can use this quantitative analysis method to conveniently judge what type of mismatch exists in the designed sensor from the measured directivity beam pattern, and easily estimate the specific magnitude of this type of mismatch, and then use this estimation to help improve the original design scheme.

## 4. Correction Algorithm for Mismatches

In the actual development process of this kind of new acoustic sensor, the ADS, it is inevitable that the differential units will be mismatched; if we can continue to use them and ensure their high directivity to some extent, it will be of great significance. In this section, a correction algorithm for the mismatches between the sensitive units of ADSs will be proposed, and both simulation results and experimental results will be used to show the correction ability of the algorithm.

### 4.1. Design of the Correction Algorithm

According to the quantitative analysis method in Section 3, the specific magnitude μ, ϕ and $\theta_{m}$ of the mismatches of the two sensitive units of an ADS can be estimated from the directivity beam pattern for a certain L/λ. Furthermore, the theoretical directivity beam pattern of an ADS with such mismatches can be obtained by substituting the estimated μ, ϕ and $\theta_{m}$ into Equation (14), the normalized directivity function with mixed mismatches. If the theoretical beam pattern with mixed mismatches is compared with the ideal cosine squared beam pattern, the difference between them can be obtained. If this difference is removed from the actually measured beam pattern, a corrected beam pattern can be obtained.

Following this idea, the correction algorithm can be divided into five steps:

1. Import and preprocess the experimental data. The preprocess is mainly to normalize the voltage data output from the experiment.
2. Determine the quantitative parameters in Table 2, which describe the mismatches.
3. Estimate the magnitude of the mismatches, μ, ϕ and $\theta_{m}$.
4. Obtain the theoretical directivity beam pattern with such mismatches.
5. Output the beam pattern after reducing the difference from the ideal shape.

Step 1 is easy to understand. The implementation of Step 2–4 is shown in Section 3.4. For Step 5, the easiest way to define the difference between the theoretical beam pattern with mixed mismatches and the ideal cosine squared beam pattern is to find their ratio. Therefore, the corrected beam pattern can be obtained using the following equation:

(21) $${NDF}_{VG\_corrected\_output}=\frac{{NDF}_{VG\_theory\_mis}}{{NDF}_{VG\_cosine\_squared}}\cdot {NDF}_{VG\_measured\_input}$$

where, ${NDF}_{VG\_corrected\_output}$ is the corrected normalized directivity function, ${NDF}_{VG\_theory\_mis}$ is the theoretical normalized directivity function with mixed mismatches obtained by Step 4 of the correction algorithm, ${NDF}_{VG\_cosine\_squared}$ is the ideal cosine squared shape normalized directivity function, and ${NDF}_{VG\_measured\_input}$ is the measured normalized directivity function before correction.

A program based on Mathematica has been developed to verify the correction ability of the algorithm according to the above algorithm steps.

### 4.2. Correction of Simulation Results

**Figure 19:**
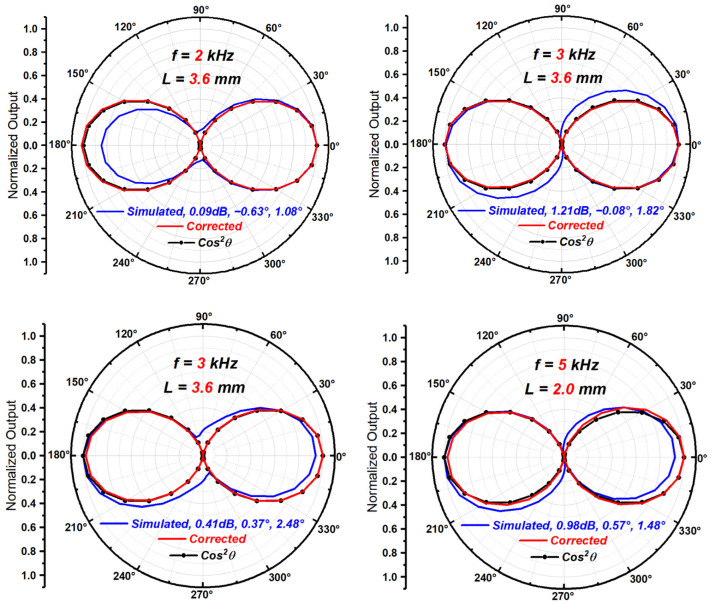
The comparison of the simulation beam patterns before and after correction.

Several groups of different spacing L and sound wave frequency f combinations with different mismatches are simulated using COMSOL’s pressure acoustics module. Additionally, the Mathematica program is used to correct the simulation results. The comparison of the directivity beam patterns before and after correction is shown in Figure 19.

As seen in Figure 19, the corrected beam patterns are more consistent with the ideal cosine squared shape than the beam patterns with mismatches before correction.

### 4.3. Correction of Experimental Results

Another ADS was developed, which is also a particle velocity type ADS based on MEMS thermal APVSs. The spacing L of the two APVS is 2.0 mm. Its directivity beam pattern were obtained at the frequencies of 4, 5 and 6 kHz. 

**Figure 20:**
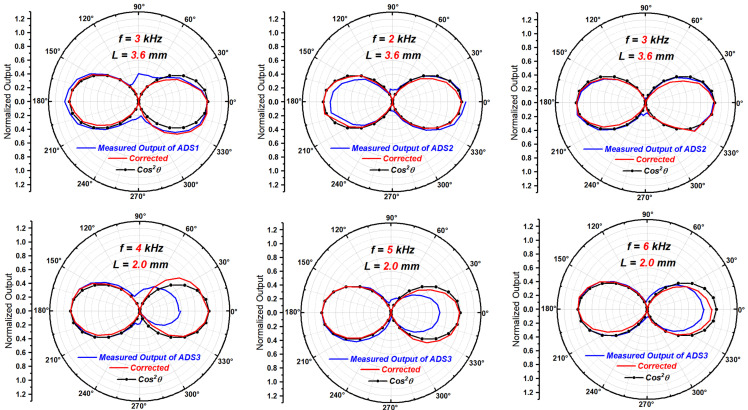
The comparison of the measured beam patterns before and after correction.

The comparison of the directivity beam patterns of the three actual ADSs mentioned in this article before and after correction is shown in Figure 20.

As seen in Figure 20, the corrected beam patterns are more consistent with the ideal cosine squared shape than the beam patterns with mismatches before correction.

For a certain mismatch, the three quantitative parameters in Table 2, ${BWI}_{VG}\left(\mu \right)$, ${DAS}_{VG}\left(\phi \right)$ and ${IAR}_{VG}\left(\theta_{m}\right)$, can be uniquely determined, according to Equations (15)–(17). Hence, in the actual use process, there is no need to perform the measured output of each test frequency from Step 1 to Step 5.

The correction of simulation results and experimental results show the ability of the designed correction algorithm for mismatches. It verifies the correctness and practicability of the theoretical model and quantitative analysis method mentioned in Section 2 and Section 3, and also provides a low-cost way for utilizing the ADSs with mismatches to continue to play their high directivity advantages.

## 5. Conclusions

In this article, the impact of different types of mismatches of the two sensitive units of an acoustic dyadic sensor (ADS), a new type of acoustic sensor with high directivity, on the directivity beam patterns were analyzed and discussed in detail from the perspectives of theory, simulation and experiment. 

A theoretical model of mixed mismatches was established based on the finite-difference approximation model of uniaxial acoustic particle velocity gradient. The change in directivity beam pattern of an ADS with only one type of mismatch was deduced based on the theoretical model of mixed mismatches. Additionally, the effects of various mismatches on the directivity beam pattern of an ADS was summarized: (1) the main effect of amplitude mismatch is to increase the beam width. (2) the main effect of phase mismatch is to create asymmetry. (3) the main effect of axial mismatch is to make the concave points bulge and sensitive axis direction deflected. After that, the change in directivity beam pattern in the presence of mixed mismatches was analyzed by providing the normalized directivity function and the directivity beam pattern. An actual ADS was demonstrated by using MEMS thermal acoustic particle velocity sensors as sensitive units. Additionally, the possible mismatches of the actual ADS can be qualitatively obtained by comparing the measured directivity beam patterns with the theoretical model of mixed mismatches. 

A quantitative analysis method based on directivity beam pattern was proposed to easily estimate the specific magnitude of the mismatches. Three quantitative parameters, Beam Width Increase (BWI), Difference of Axial Sensitivity (DAS) and Ideal Axial Ratio (IAR) were used or defined with equations based on the theoretical model of mixed mismatches proposed in this article. An actual ADS with spacing of 3.6 mm based on MEMS thermal APVS chips was developed to be used as an example to illustrate the usage of this quantitative analysis method. By using the three quantitative parameters, BWI, DAS and IAR, the magnitude of different types of mismatches, μ, ϕ and $\theta_{m}$, were successfully estimated. Additionally, the theoretical beam patterns with these mismatches were basically consistent with the measured results. Therefore, this method can be used to help the design of ADSs, which is the new type of high directivity acoustic sensor.

Lastly, a correction algorithm based on the theoretical model of mixed mismatches and quantitative analysis method proposed in this article, for the mismatches between sensitive units of ADSs, was successfully demonstrated to correct several groups of simulated and measured beam patterns with mixed mismatches. 

However, there are still differences between the correction beam patterns and the ideal cosine squared shape, especially for the correction of the measured results. Therefore, the correction algorithm proposed in this article is not optimal. From the experimental point of view, it may be because the position of the sensor is not completely fixed during the measurement process, and the test sound field is not a perfect plane wave. It might be improved by improving the fixture and testing in an anechoic chamber. From the point view of the algorithm, there are two possible ways to improve the algorithm in future research: (1) optimize or redefine the three quantitative parameters (BWI, DAS and IAR), so that they can represent the deviation of directivity beam patterns more accurately; and (2) find a more accurate method to define the difference between the theoretical beam pattern with mixed mismatches and the ideal cosine squared beam pattern.

## Figures and Tables

**Table 1:** The main effects of the three types of mismatches on the directivity beam pattern of an ADS.

Amplitude Mismatch μ	Phase Mismatch ϕ	Axial Mismatch $\;\theta_{m}$
Increase the beam width	Create asymmetry	Make the concave points bulge and sensitive axis direction deflected

**Table 2:** Quantitative parameters of an ADS’s directivity beam pattern to describe the mismatches.

Amplitude Mismatch μ	Phase Mismatch ϕ	Axial Mismatch $\;\theta_{m}$
Beam Width Increase (BWI) ${BWI}_{VG}\left(\mu \right)$	Difference of Axial Sensitivity (DAS) ${DAS}_{VG}\left(\phi \right)$	Ideal Axial Ratio (IAR) ${IAR}_{VG}\left(\theta_{m}\right)$

**Table 3:** Estimated magnitude of mismatches of the ADSs from Figure 10 and [27].

	Amplitude Mismatch μ	Phase Mismatch ϕ	Axial Mismatch $\;\theta_{m}$
The ADS1 from Figure 10 L = 3.6 mm, f = 3 kHz	${BWI}_{VG\_actual}=0.0779$	${DAS}_{VG\_actual}=0.566$	${IAR}_{VG\_actual}=13.257$
	μ≈1.048 (0.41 dB)	ϕ≈0.374°	$\theta_{m}\approx 2.481{}^{\circ}$
The ADS2 from [27] L = 3.6 mm, f = 3 kHz	${BWI}_{VG\_actual}=0.459$	${DAS}_{VG\_actual}=-0.116$	${IAR}_{VG\_actual}=15.936$
	μ≈1.151 (1.21 dB)	ϕ≈−0.0768°	$\theta_{m}\approx 1.823{}^{\circ}$

## Data Availability

The data presented in this study are available on request from the corresponding author. The data are not publicly available due to restrictions of undergoing projects.
